# Marine Bioactive Compounds as Nutraceutical and Functional Food Ingredients for Potential Oral Health

**DOI:** 10.3389/fnut.2021.686663

**Published:** 2021-12-02

**Authors:** Yi-Zhen Huang, Zheng Jin, Zhe-Ming Wang, Li-Bo Qi, Shuang Song, Bei-Wei Zhu, Xiu-Ping Dong

**Affiliations:** ^1^School of Food Science and Technology, Academy of Food Interdisciplinary Science, Dalian Polytechnic University, Dalian, China; ^2^National Engineering Research Center of Seafood, Collaborative Innovation Center of Seafood Deep Processing, Liaoning Province Collaborative Innovation Center for Marine Food Deep Processing, School of Food Science and Technology, Dalian Polytechnic University, Dalian, China

**Keywords:** marine bioactive compounds, oral health, dental caries, chewing gum, functional food

## Abstract

Oral diseases have received considerable attention worldwide as one of the major global public health problems. The development of oral diseases is influenced by socioeconomic, physiological, traumatic, biological, dietary and hygienic practices factors. Currently, the main prevention strategy for oral diseases is to inhibit the growth of biofilm-producing plaque bacteria. Tooth brushing is the most common method of cleaning plaque, aided by mouthwash and sugar-free chewing gum in the daily routine. As the global nutraceutical market grows, marine bioactive compounds are becoming increasingly popular among consumers for their antibacterial, anti-inflammatory and antitumor properties. However, to date, few systematic summaries and studies on the application of marine bioactive compounds in oral health exist. This review provides a comprehensive overview of different marine-sourced bioactive compounds and their health benefits in dental caries, gingivitis, periodontitis, halitosis, oral cancer, and their potential use as functional food ingredients for oral health. In addition, limitations and challenges of the application of these active ingredients are discussed and some observations on current work and future trends are presented in the conclusion section.

## Introduction

Oral and periodontal diseases can determine severe functional, phonatory and aesthetic impairments and are the main cause of adult tooth loss (1). According to statistics, the number of people suffering from untreated dental caries, severe periodontitis and oral diseases has reached 3.5 billion worldwide (2, 3). Moreover, the number of cancers occurring in the lips and oral cavity reached 377,713 in 2020, based on GLOBOCAN statistics (4). Oral diseases have become a major global public health problem. The growing awareness of the oral health benefits has compelled science and industry to conduct research on nutrients for the prevention and treatment of oral diseases, including isolated nutrients and compounds as dietary supplements. The marine-sourced compounds include protein and peptides, protein, ω-3 polyunsaturated fatty acids (PUFA), polysaccharides, polyphenolic compounds, enzymes, vitamins and pigments (5) with a wide range of physiological activities such as antitumor, antioxidant, antiviral, and immunomodulatory activities (6, 7). Thus, the wide variety of marine species is not only a source of important active compounds for the treatment and prevention of various diseases, but also a potential source of ingredients for oral health maintenance (8–10).

**Figure 1:**
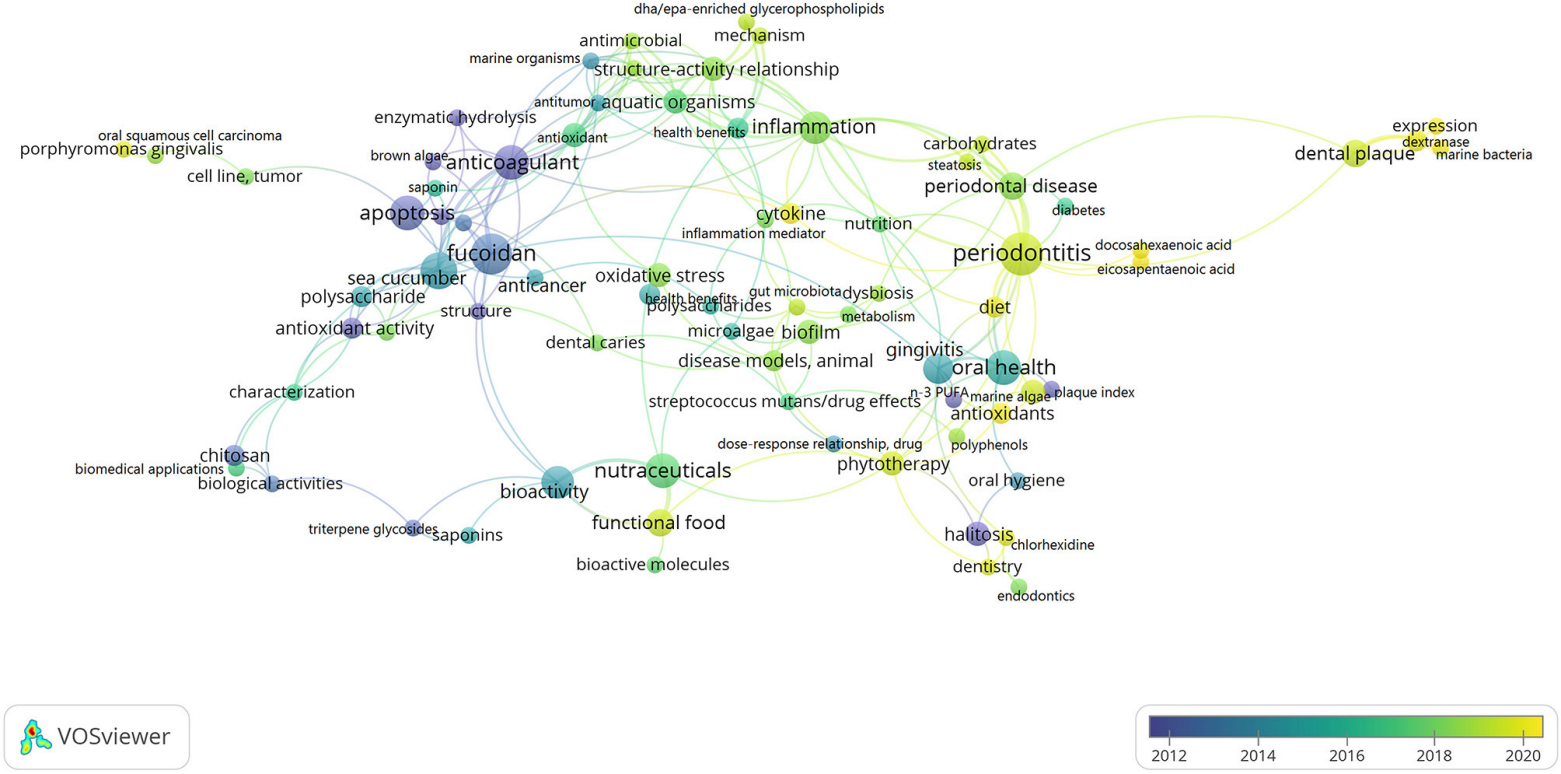
Overlay visualization of keyword statistics.

Indeed, bioactive materials and molecules from marine sources have been applied in many potential research areas in dental science. Shaikh et al. (11) conducted a review on the application of marine derived ingredients such as chitosan, bio-adhesives, tissue regeneration gel, calcium hydroxyapatite, gypsum and algal extracts in the field of dentistry. Ibrahim et al. (12) found that marine bioactive ingredients have inhibitory effects on *Streptococcus mutans* (*S. mutans*) and can be used to maintain dental health. In recent years, various scientific research publications on marine bioactive ingredients health benefits and the development of marine nutraceuticals have been published. Two hundred and ninety-eight papers related to marine bioactive compounds and oral disease published in the past 18 years were retrieved from Science Direct and analyzed by Vosviewer for keywords (marine bioactive compounds, oral disease/disorder/health). The results of the co-occurrence analysis showed that the research related to the effects of marine bioactive compounds on oral health gradually shifted to periodontitis, dental plaque, phytotherapy and functional food. Figure 1 shows the overlay visualization of keyword statistics.

Published reviews with a comprehensive description of marine bioactive compounds as nutritional and functional food ingredients for potential oral health are limited. Therefore, it is necessary to systematically summarize the benefits, risks and prospects of marine bioactive compounds in oral health applications. This review provided a comprehensive overview of different marine bioactive compounds and their health benefits, as well as their potential to be used as functional food ingredients for oral health. In addition, the limitations and challenges of the application of these active ingredients were discussed and some perspectives on the current work and future trends were presented in the conclusion section.

## Oral Health

### Oral Health, Plaque and Microbiome

Oral health is an important indicator of people's overall health and quality of life. The World Health Organization (WHO) defines oral health as “a state of being free from chronic mouth and facial pain, oral and throat cancer, oral infection and sores, periodontal (gum) disease, tooth decay, tooth loss, and other diseases and disorders that limit an individual's capacity in biting, chewing, smiling, speaking, and psychosocial well-being” (13, 14). Recent years, oral epidemiology is shifting globally and the main oral disorders that plague people's lives include: dental caries, periodontal disease, oral cancer, oral manifestations of HIV, oro-dental trauma, cleft lip and palate, and Noma (13, 14). Oral diseases can lead to pain, impaired function and reduced quality of life, which can have a direct impact on an individual's quality of life (15).

**Figure 2:**
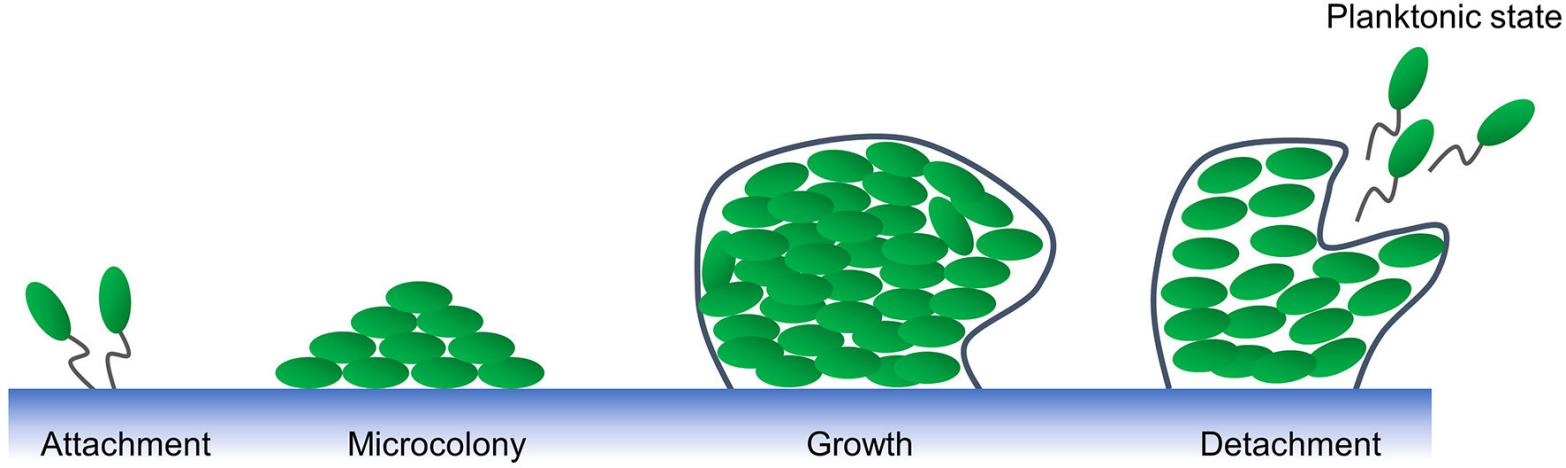
Biofilm formation.

Plaque in the oral cavity is considered to be the basis for the initiation and development of dental caries and periodontitis. Plaque consists of a large number of bacteria, intercellular material, a small amount of white blood cells, shed epithelial cells and food debris, and is a bacterial biofilm that cannot be washed away by water. In dentistry, biofilms are defined as microbial communities of surface-attached cells embedded in a self-produced extracellular matrix (16–19). Dental plaque biofilms on the tooth surface form a bacterial envelope that is coordinated and balanced with the surrounding tissues (16). The development of biofilm formation was shown in Figure 2 (16, 17). Dental plaque biofilms have the ability to interact in a complex interplay with the innate immune and inflammatory networks that ensure the maintenance of the host's health (20).

Indeed, the development and progression of oral disease is largely influenced by oral microbiome in the oral cavity (21–26). A variety of microorganisms collectively influence the microecological balance of the oral cavity through symbiotic “competitive” antagonism (15). Many of these microorganisms are harmless; a few, such as S. *mutans, Actinomyces sp., Treponema denticola, Tannerella forsythia, Bacteroides sp., A. actinomycetemcomitans, Staphylococcus intermedius, Porphyromonas gingivalis* and *Candida albicans*, can cause oral infections (15). Moreover, factors such as immunity weakening or steroid treatment can disturb the balance of oral bacteria and can also cause opportunistic infections. Nutritional deficiencies can also alter the composition of the oral microbiome and lead to oral health problems (27). Any process resulting in weakened immunity or a factor such as steroid therapy that disturbs the oral bacterial homeostasis can cause opportunistic infections (28). Deficient diet can also alter the composition of the oral microbiome leading to oral diseases such as dental caries and periodontitis. Therefore, to ensure oral health, the oral cavity needs regular care and maintenance.

### Oral Health Maintenance

Mechanical oral hygiene aid is an important way to maintain oral health (29). The most common mechanical oral cleaning is toothbrushing, but this method is not appropriate for removing plaque from the gingival sulcus of normal dentition (30, 31). Hence, regular toothbrushing needs to be accompanied by some supplementary modalities for plaque removal. Mouth rinses and chewing gum are oral cleaning aids used in the daily routine (14, 32, 33). Rinses wash the mouth quickly by mechanically flushing, making it impossible for plaque to accumulate on the teeth. Anti-inflammatory compounds such as chlorhexidine, dextranase, menthol, and triclosan are used in the treatment of oral diseases, but can lead to side effects with long-term use (34). Calvo-Guirado et al. (35) compared the effectiveness of 0.20% chlorhexidine with Sea 4® Encias (seawater) oral rinse. Based on this study, the use of seawater mouthwash had similar antimicrobial activity to chlorhexidine. Despite the better clinical performance of chlorhexidine, seawater mouthwash can reduce side effects such as tooth staining and become a daily option for maintaining oral health. Chewing gum, on the other hand, increases the secretion of saliva in the mouth through the mechanical stimulation of chewing motion, rinsing the mouth and removing of dietary fermentable carbohydrates and plaque from the mouth. Moreover, the base of chewing gum is highly sticky, which further removes plaque from the teeth. Although sugar-free chewing gum can be used as an adjunct to brushing and can significantly and slightly reduce plaque production, there is no significant improvement in gingivitis (36). Based on these characteristics, if functional ingredients with the ability to resist *S. mutans* strains [e.g., quercetin (Qt)] are added to chewing gum formulations, chewing gum may be used as a delivery and retention of bioactive molecules in a particularly effective way, with potential anti-caries effects. Besides cleaning, the central role of diet, natural agents and nutrition is also considered to be indirectly responsible for the health of periodontal tissues and the fight against resorption of alveolar bone resorption (27, 37).

## Research Progress on Marine Bioactive Compounds in Oral Health

With the improvement of biological separation and purification technology and the in-depth research of marine biochemical drugs, an increasing number of marine bioactive substances have been isolated and identified (38–49). Now, more than 4,000 marine bioactive compounds have been isolated, but only a small number of them have been intensively studied and exploited (50). In terms of chemical structure, marine bioactive substances mainly include peptides, polysaccharides, alkaloids, terpenoids, macrocyclic polyesters, polyethers, polyenes, unsaturated fatty acids and steroids (6, 7, 51, 52). Table 1 summarizes the various marine bioactive ingredients used to prevent and treat oral diseases ranging from dental caries to halitosis.

**Table 1:** List of marine active ingredients for various oral diseases and their causative pathogens.

**Objective**	**Compounds**	**Form**	**Source**	**Results**	**References**
Antibacterial	*Chlorella vulgaris* and *Dunaliella salina* extract	/	/	The biofilms of *Streptococcus mutans* were more effectively prohibited by *D. salina* extract than *C. vulgaris* extract.	(51)
Articular cartilage regeneration using human dental pulp stem cells cultured	Alginate	Hydrogel	/	Dental pulp stem cells (hDPSCs) cultured in 3% alginate hydrogels may be useful for regeneration of articular cartilage.	(52)
Dental caries	Dextranase	/	Marine bacterium *Catenovulum sp*. (Cadex)	Cadex from a marine bacterium was shown to be an alkaline and cold-adapted endo-type dextranase suitable for development of a novel marine agent for the treatment of dental caries.	(53)
Dental plaque	Dextranase	/	*Catenovulum agarivorans* MNH15	Dextranase has high application potential in dental products such as toothpaste and mouthwash.	(54)
Dental plaque	Polysaccharide extract of sea cucumber *Stichopus horrens*	/	/	Polysaccharide extract of sea cucumber *S. horrens* had the potential to be further expanded into a beneficial substance with therapeutic feature that could be used in preventive and restorative dentistry	(11)
Dental pulp biomineralization and Differentiation	Alginate	Alginate/Hydroxyapatite-Based Nanocomposite Scaffolds	/	Alg/HAp scaffolds as feasible composite materials in tissue engineering, being capable of promoting a specific and successful tissue regeneration as well as mineralized matrix deposition and sustaining natural bone regeneration.	(55)
Dental pulp repair	Alginate	Hydrogel	/	Alginate hydrogels provide an appropriate matrix in which dental regeneration can take place and may also be useful for delivery of bioactive molecules, such as growth factors, to enhance the natural regenerative capacity of the dental pulp.	(56)
Encapsulation of periodontal ligament (PDLSCs) and gingival mesenchymal (GMSCs) stem cells system	Alginate	Hydrogel	/	Alginate is a promising candidate as a non-toxic scaffold for PDLSCs and GMSCs. It also has the ability to direct the differentiation of these stem cells to osteogenic and adipogenic tissues as compared to the control group *in vitro*.	(57)
Gingivitis	Sea cucumber extract	Toothpastes	/	Toothpaste containing sea cucumber extract produced statistically significant reduction in gingival inflammation.	(58)
Gingivitis	n-3 PUFA	/	/	n-3 PUFA induced a tendency toward reduced inflammation but it was not possible to conclude significant efficacy.	(59)
Gingivitis	Fucoidan	/	Brown algae (*Fucus vesiculosus (F85), Fucus vesiculosus (F95), Macrocytis pyrifera (M85), Undaria pinnatifida (U95), Hizikia fusiforme, Kjellmaniella crassifolia, Laminaria japonica, Sargassum honeri, Undaria pinnatifida*), Green algae (*Capsosiphon fulvescens, Codium fragile*), Red alga (*Grateloupia flilicina*)	The fucoidan at the concentrations of above 250 μg mL^−1^ completely suppressed the biofilm formations and planktonic cell growths of *S. mutans* and *S. sobrinus*.	(60)
Gingivitis	*Enteromorpha linza* extract	Mouth rinse	/	The twice-daily use of an *E. linza* extract mouth rinse can inhibit and prevent gingivitis.	(61)
Halitosis	Three phlorotannins (eckol, dioxinodehydroeckol and dieckol)	/	Brown seaweed *Eisenia bicyclis*	Phlorotannins derived from *E. bicyclis* can be an effective deodorizing constituent in the food industry and pharmaceutical industries	(62)
Inducing mineralization of dental implants	Alginate	Poly-l-lysine/Sodium alginate coating	/	The composite coating could prevent bacterial infections and facilitate mineralization *in vivo* in the early postoperative period, and then, the mineralized surface could enhance the cytocompatibility	(63)
OSCC	*Enteromorpha compressa* solvent extracts	/	/	The presence of novel bioactive compounds in *E. compressa* has uncovered possible therapeutic value against OSCC by modulating antioxidant defense system, apoptosis and autophagy that could be used to explore very competent algal candidates for the development of potential alternative anticancer drugs.	(64)
OSCC	Prodigiosin (PG)	/	Alkaloid and natural red pigment as a secondary metabolite of *Serratia marcescens*	PG under various concentrations and time courses were shown to effectively cause cell death and cell-cycle arrest in OECM1 and SAS cells.	(65)
OSCC	Sandensolide	/	*Sinularia flexibilis*	Both the *in vitro* bioassay and the zebrafish xenograft model demonstrated the anti-oral cancer effect of sandensolide.	(66)
OSCC	11-dehydrosinulariolide	/	Soft coral *Sinularia leptoclados*	Treatment with 11-dehydrosinulariolide for 6 h significantly induced both early and late apoptosis of CAL-27 cells, observed by flow cytometric measurement and microscopic fluorescent observation.	(67)
OSCC	Pardaxin	/	*Pardachirus marmoratus*	Pardaxin shows antibacterial and antitumor activities. However, pardaxin-induced inhibition of oral cancer and the mechanism of tumor reduction in buccal pouch carcinogenesis after pardaxin painting remain undetermined.	(68)
Periodontitis	Sulfated polysaccharides (PLS)	/	Marine algae (*genus Gracilaria*)	The adjunct treatment with PLS from *Gracilaria caudata* could prevent the periodontal and hepatic tissue alteration caused by periodontitis.	(69)
Periodontitis	Chitosan	Drug delivery system (LDDS) with chitosan and poly vinyl alcohol (PVA)	/	CM-chitosan microsphere (Cs2-Ms) had better potentials used as core parts of the novel designed LDDS in the future developments.	(70)
Periodontitis	Sea cucumber extract	/	/	sea cucumber extract has an effect on periodontitis and can be an alternative to treating inflammation	(71)
Periodontitis	Omega-3 polyunsaturated fatty acids (PUFA)	/	/	Dietary intervention with high-dose of omega-3 PUFA during non-surgical therapy may have potential benefits in the management of periodontitis.	(72)
Periodontitis	Omega-3 polyunsaturated fatty acids (PUFA)	/	/	Both DHA and EPA have significant antimicrobial activity against the six bacterial species (*Streptococcus oralis, Actinomyces naeslundii, Veillonella parvula, Fusobacterium nucleatum, Porphyromonas gingivalis, and Aggregatibacter actinomycetemcomitans*) included in this biofilm model.	(73)
Periodontitis	Sulfated polysaccharides (PLS)	/	Marine algae of the *genus Gracilaria*	The adjunct treatment with PLS from *G. caudata* could prevent the periodontal and hepatic tissue alteration caused by periodontitis.	(69)
Supragingival calculus, plaque formation, and gingival health	Alga (*ascophyllum nodosum*)	/	/	Fifty-two participants showed less calculus formation in the alga group than in the control group. Plaque (*p* = 0.008) and gingival bleeding (*p* = 0.02) were also significantly less in the alga group. However, no significant difference was found between the groups for gingivitis (*p* = 0.13).	(74)

### Delay the Development of Dental Caries by Inhibiting *S. mutans*

Dental caries, often referred to as cavities or tooth decay, can be defined as the localized chemical dissolution of tooth surfaces due to an imbalance in the oral microbial community (15, 53). According to the Global Oral Health Data Bank, the global prevalence of dental caries ranges from 49 to 83% (54), with ~486 million children suffering from primary caries and 2.4 billion children suffering from permanent caries (55). *S. mutans*, the main causative agent in the development of dental caries, has the ability to synthesize extracellular glucans exclusively from dietary sucrose (56–58). The main marine bioactive ingredients that are currently used to slow down the development of dental caries by inhibiting the development of *S. mutans* are alginate, sulfated polysaccharides, microalgae extracts, and *glucanases* from marine bacteria. These bioactive components inhibit the development of *S. mutans* by reducing sugar utilization, antibacterial and eliminating biofilms.

Alginate is used to slow down the development of dental caries by reducing the use of sucrose by *S. mutans*. As a non-reducing sugar extracted from seaweed, alginate can be added to foods as a sucrose substitute to slow down the development of dental caries (59). However, alginate did not completely prevent the development of dental caries and showed no effect in disrupting the biofilm formation of *S. mutans* (60). In contrast, fucoidan (*Fucus vesiculosus* F85) possessed notable antimicrobial activities against *S. mutans*, but no biofilm elimination was observed in the study of Jun et al. (61). Microalgae such as *Dunaliella salina* and *Chlorella vulgaris* have antimicrobial activity and are widely used in food, coloring and dietary supplements. Jafari et al. (62) showed that certain concentrations of *C. vulgaris* and *D. salina* extracts had the ability to inhibit not only *S. mutans* but also biofilm formation. The mechanism for the anti-biofilm activity of *C. vulgaris* and *D. salina* may be due to the glucosyltransferase (GTF) inhibition of *C. vulgaris* and *D. salina* extracts activity, which prohibits the biosynthesis of water-insoluble glucans.

Dextranase from marine bacteria are also known to prevent biofilm formation in *S. mutans*. Ren et al. (63) showed that Cadex, an alkaline and cold-adapted endo-type dextranase from marine bacteria, impeded the formation of *S. mutans* biofilms to some extent by specifically cleaving the α-1,6 glycosidic bond. Meanwhile, Lai et al. (64) screened strains of *Catenovulum agarivorans* MNH15 from marine samples, which produced a dextranase with stable *S. mutans* inhibition, and its activity was not affected by sodium fluoride, xylitol, and sodium benzoate in dental care products. Therefore, more marine bacterial dextranases can be explored for the development of oral care products or novel marine agents for the treatment of dental caries to meet the vast demand for caries prevention.

### Managing Gingivitis: Compounds, Underlying Mechanism

Gingivitis is one of the most common periodontal diseases, and clinically gingivitis can be divided into two categories (29, 65): (1) gingival diseases caused by non-plaque biofilms, which do not resolve even after plaque removal and may manifest as a systemic disease or in the local oral cavity. (2) Another class is plaque-induced gingivitis, caused by the interaction of plaque biofilm and the host's immune response. The infection remains contained in the gingiva and does not extend to the periodontium, but it can be reversed by reducing plaque at the gingiva and root canals (15, 65). Plaque-induced gingivitis has many clinical features and is mainly predicated on the presence or absence of bleeding at the time of probing. Hence, the main parameters for the determination of gingivitis are GI (Gingival Index) and BOP (Bleeding on probing) (65). The main marine bioactive ingredients that have been reported for anti-gingivitis are sea cucumber extract, algae extract and n-3 polyunsaturated fatty acids (PUFAs).

Research by Wen (66) showed that toothpastes containing sea cucumber extracts were effective in reducing gingivitis. Moreover, sea cucumber *Stichopus horrens* (SH) extracts can promote gingival tissue healing in addition to its anti-streptococcal ability. A study by Bakar et al. (67) showed that a 9% concentration of SH-containing toothpaste (SHCT) was effectively in reducing gingival inflammation (14 days of continuous use) and probe bleeding (30 days of continuous use). The anti-inflammatory effect was also observed with *Enteromorpha linza*, a green algae whose extract strongly inhibited the growth of *Prevotella intermedia* and *Porphyromonas gingivalis* (68). Cho (68) determined that the anti-inflammatory effect of *E. linza* was reduced by plaque index (PI), GI, and BOP measured the clinical effect of mouthwash with *E. linza* extract on gingivitis disease. The results indicated that mouthwash containing *E. linza* extract had a significant inhibitory effect on gingival abscesses *P. gingivalis* and *P. intermedia*, had a significant reduction in plaque, improved gingival tissues, and reduced bleeding. Hence, the measure of using *E. linza* extract mouthwash twice daily was recommended by this clinical study to manage gingivitis.

**Figure 3:**
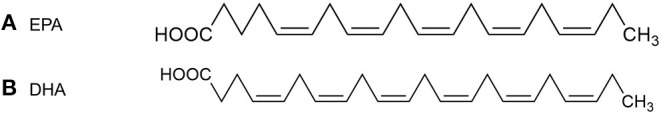
The chemical structure of **(B)** DHA and **(A)** EPA.

Furthermore, the anti-inflammatory effects of n-3 PUFAs from marine organisms such as *eicosapentaenoic acid* (EPA) and *docosahexaenoic acid* (DHA) (Figure 3) on gingiva have been demonstrated in animal models. Campan et al. (69) showed that n-3 PUFAs was able to induce a trend toward reduced inflammation. This is because EPA may show anti-inflammatory effects by competitively inhibiting the pro-inflammatory cyclooxygenase-1/2 (COX-1/2), forming PGE2 and producing PGE3. Notably, DHA inhibits *lipoxygenase* (LOX) to produce the pro-inflammatory COX metabolite D-series aminotransferases, thus exhibiting a more potent inhibitory effect on inflammation than EPA. Moreover, n-3 PUFAs has antimicrobial activity. Ribeiro-Vidal et al. (70) demonstrated that DHA and EPA were more effective in this biofilm model of *Streptococcus oralis, Actinomyces naiveus, Veillonella parvula, Fusobacterium nucleatum, P. gingivalis*, and *Aggregatibacter actinomycetemcomitans* all exhibited significant antibacterial activity (71).

### Periodontitis: Compounds, Primary Prevention, Underlying Mechanisms

Periodontitis is a chronic multifactorial inflammatory disease that may result from a host immune response triggered by bacterial biofilms and is characterized by inflammation of gingival tissue and resorption of alveolar bone (20, 65, 72). Periodontal disease can be treated with non-surgical as well as surgical therapies. Both supra- and subgingival plaque and calculus removal should be performed as an initial stage of the treatment of periodontal disease (65). Common pathogens of periodontitis are *Treponema denticola, P. gingivalis, Tannerella forsythia, Bacteroides sp., Actinomyces sp, Staphylococcus intermedius, Actinomyces actinomycetemcomitans*, and *Candida albicans* (72, 73). The main marine bioactive ingredients that have been reported to be beneficial for periodontal health are chitosan, sea cucumber extract, n-3 PUFAs, and algae extracts.

**Figure 4:**
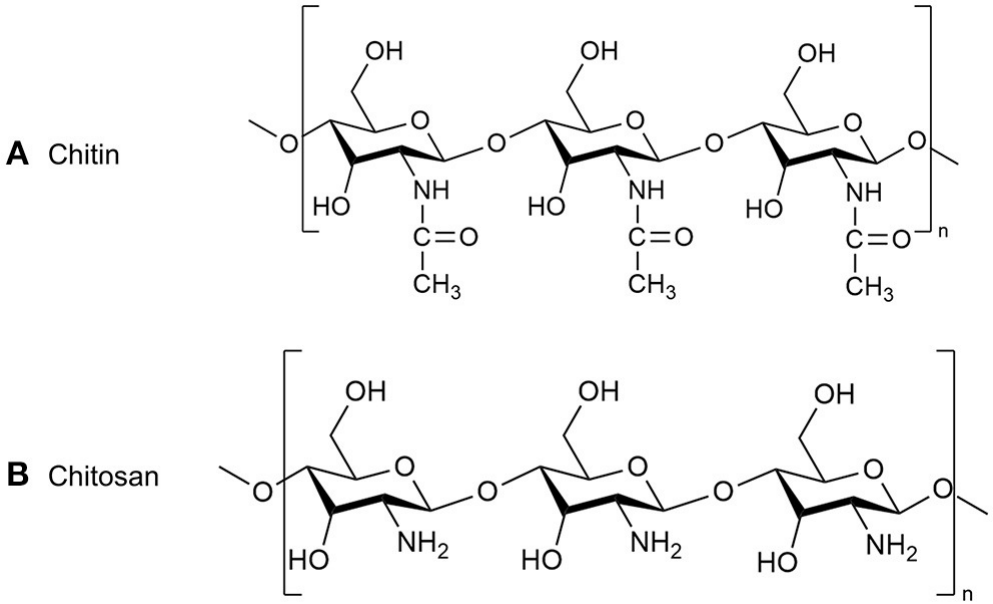
The chemical structure of **(A)** chitin and **(B)** chitosan.

**Figure 5:**
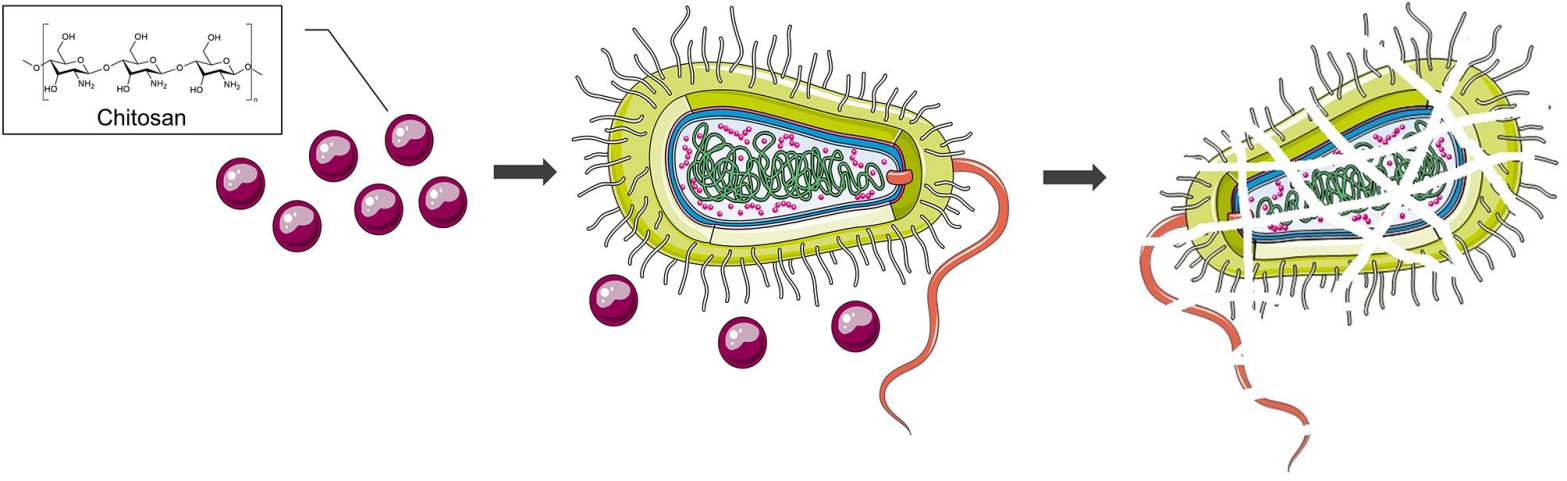
Inhibition of bacterial activity by chitosan.

Chitosan is a chemically processed form of chitin (Figure 4) with the ability to *S. mutans, A. actinomycetemcomitans* and on *P. gingivalis* (74–76). Choi et al. (77) evaluated the *in vitro* antimicrobial properties of a chitosan (Figure 5) (72). A novel formulation based on the combination of chitosan and poly vinyl alcohol (PVA) was prepared by Wang et al. (78). Both *in-vivo* and *in-vitro* experiments demonstrated that the CM-chitosan microsphere (Cs2-Ms) is soft, more hydrophilic, and rapidly degradable by diffusion under physiological conditions. Besides, the authors pointed out that in the future, Cs2-Ms have more potential than Cs1-Ms to be used as core component of newly designed localized drug delivery system (LDDS) for the treatment of periodontitis. Sea cucumber extracts have been shown to have antibacterial effects and the compounds they contain with potential anti-inflammatory effects are saponins, which reduce the activity of COX-2, which plays a role in stimulating inflammatory mediators (12, 79–81). Hence, sea cucumber has a role in periodontitis and can be used as an alternative treatment for inflammation (81).

**Figure 6:**
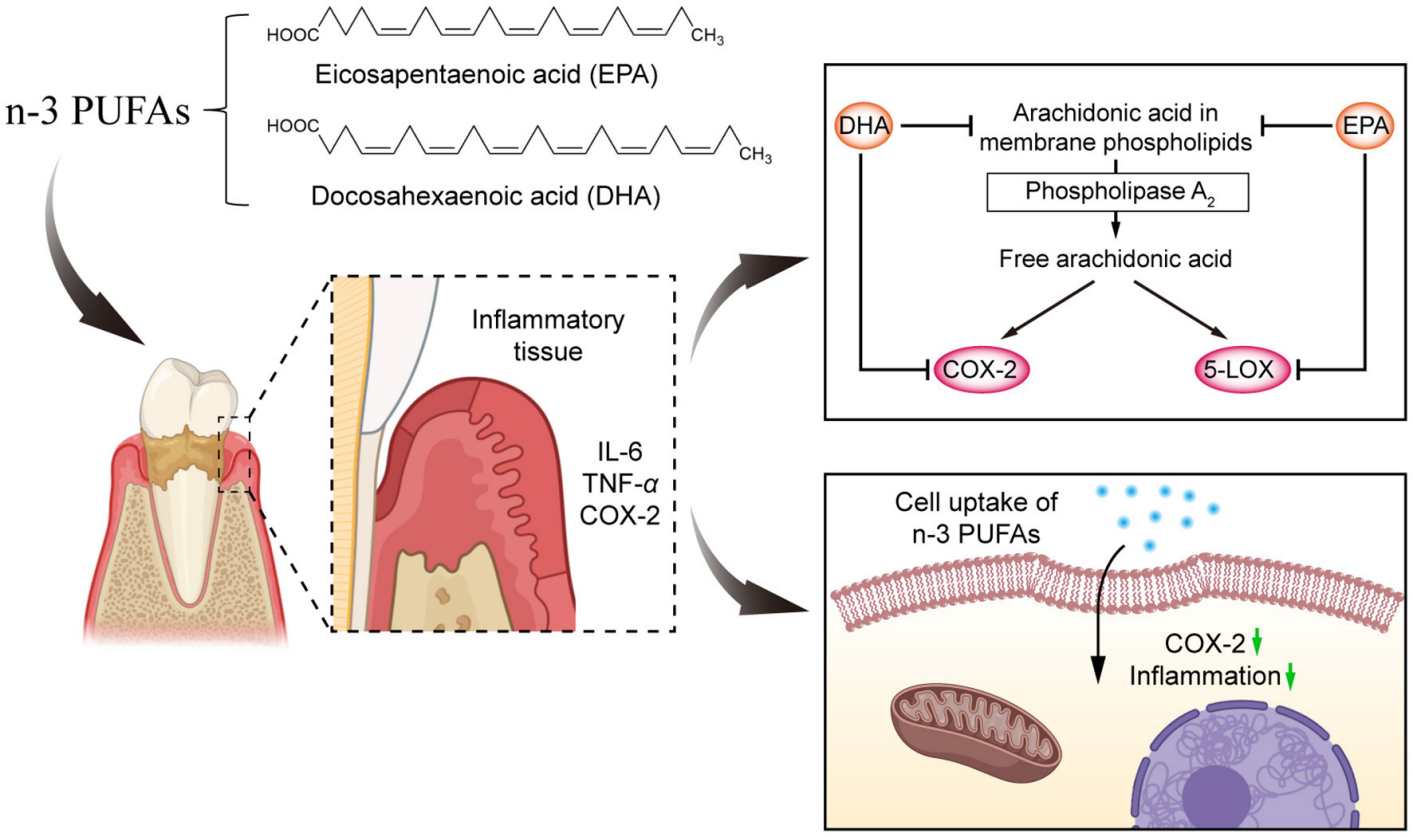
The role of n-3 PUFAs in the reduction of periodontitis.

N-3 PUFAs has antibacterial and anti-inflammatory activity, which can be used for the prevention and treatment of periodontitis (82). Ribeiro-Vidal et al. (70) indicated that the results of DHA and EPA showed significant antimicrobial activity in the biofilm model of *Streptococcus oralis, Actinomyces naeslundii, V. parvula, Fusobacterium nucleatum, P. gingivalis*, and *Aggregatibacter actinomycetemcomitans* (71). As shown in Figure 6, the invasion of bacteria and their products into periodontal tissues leaded to the production of inflammatory mediators and enzymes such as IL-6, TNF-α, COX-2, etc. N-3 PUFAs were able to reduce the tendency of inflammation by competitively inhibiting the production of COX-2 and LOX (83). A study by Stańdo et al. (83) suggested that dietary intervention with high doses of n-3 PUFAs during non-surgical treatment may be helpful in the periodontitis treatment. By evaluating dietary intervention in periodontitis patients (stage III and IV), this study revealed that after 3 months of treatment, saliva samples from patients receiving n-3 PUFAs intervention showed significantly higher levels of the anti-inflammatory *interleukin-10* and significantly lower levels of the pro-inflammatory *cytokines interleukin-8* and *interleukin-17* (83). Kruse et al. (84) used meta-analysis to evaluate the effect of n-3 PUFAs in periodontal treatment with adjunctive administration. The result showed that n-3 PUFAs appeared to have a positive effect on periodontal wound healing in terms of reduced clinical attachment level (CAL) and probing depth (PD). Based on this study, patients receiving periodontal therapy may benefit from nutritional counseling.

Marine algae have recently gained popularity for their bioactive molecules and their oral applications. For example, sulfated polysaccharides (PLS) has been reported to have anti-inflammatory effects and to prevent periodontal and liver tissue changes caused by periodontitis (85). *Ecklonia cava*, an edible marine brown algae, whose ethanol extract (ECE) can cause tumor necrosis factor-α (TNF-α), interleukin-1β (IL-1β) and interleukin-6 (IL-6) gene downregulated (86, 87). An Indian red alga *Kappaphycus alvarezii* (KAB) is rich in marine bromophenols (MBs), which can downregulate the mRNA level in the gene of *P. gingivalis*, and has an inhibitory effect on it. Additionally, KAB has been reported to inhibit gingipain and hemagglutination activities. As a potent natural metabolite, MBs has potential for use in dental products (88). *Spirulina maxima* has also been reported to be biologically active against periodontitis. Kang et al. (89) indicated that in the presence of *S. maxima*, inflammatory cytokines such as TNF-α, IL-1β, IL-6, and the inflammatory transcription factor NF-κB were reduced in gingival tissues, the expression of myeloperoxidase (MPO) activity and matrix metallopr oteinases (MMPs) was decreased, and osteogenic-related factors were promoted. Thus, this study showed that *S. maxima* reduces gingivitis-induced periodontitis and consequently bone loss through anti-inflammatory effects (89).

### Oral Squamous Cell Carcinomas: Compounds, Underlying Mechanisms

The most common oral cancers are found in the oral cavity and include the lips, tongue, gums, oral mucosa, floor of the mouth, hard palate, maxilla, mandible, nasopharynx, oropharynx, and hypopharynx (90). More than 90% of oral cancers are squamous cell carcinomas (91). It is estimated that 657,000 new cases of oral cavity and throat cancer are diagnosed each year, with more than 330,000 deaths (92). There are many factors that contribute to oral cancer. Besides tobacco, heavy consumption of alcoholic beverages, inappropriate dietary habits, poor oral hygiene, chronic inflammatory processes in the oral cavity, various viruses and bacteria, dentures, mucosal trauma, and mucosal trauma from restorations are also risk factors for the development of oral squamous cell carcinoma (OSCC) (93, 94). *P. gingivalis* causes oral inflammation and plays an important role in the development of OSCC. This is because *P. gingivalis* is able to convert nitrite in saliva to nitric oxide (NO), a metabolite that regulates different cancer-related manifestations such as apoptosis, cell cycle, angiogenesis, invasion and metastasis (68, 95). Therefore, early diagnosis and treatment of periodontitis is not only beneficial for the maintenance of patients' oral health, but also important for the prevention of OSCC (68, 96).

**Figure 7:**
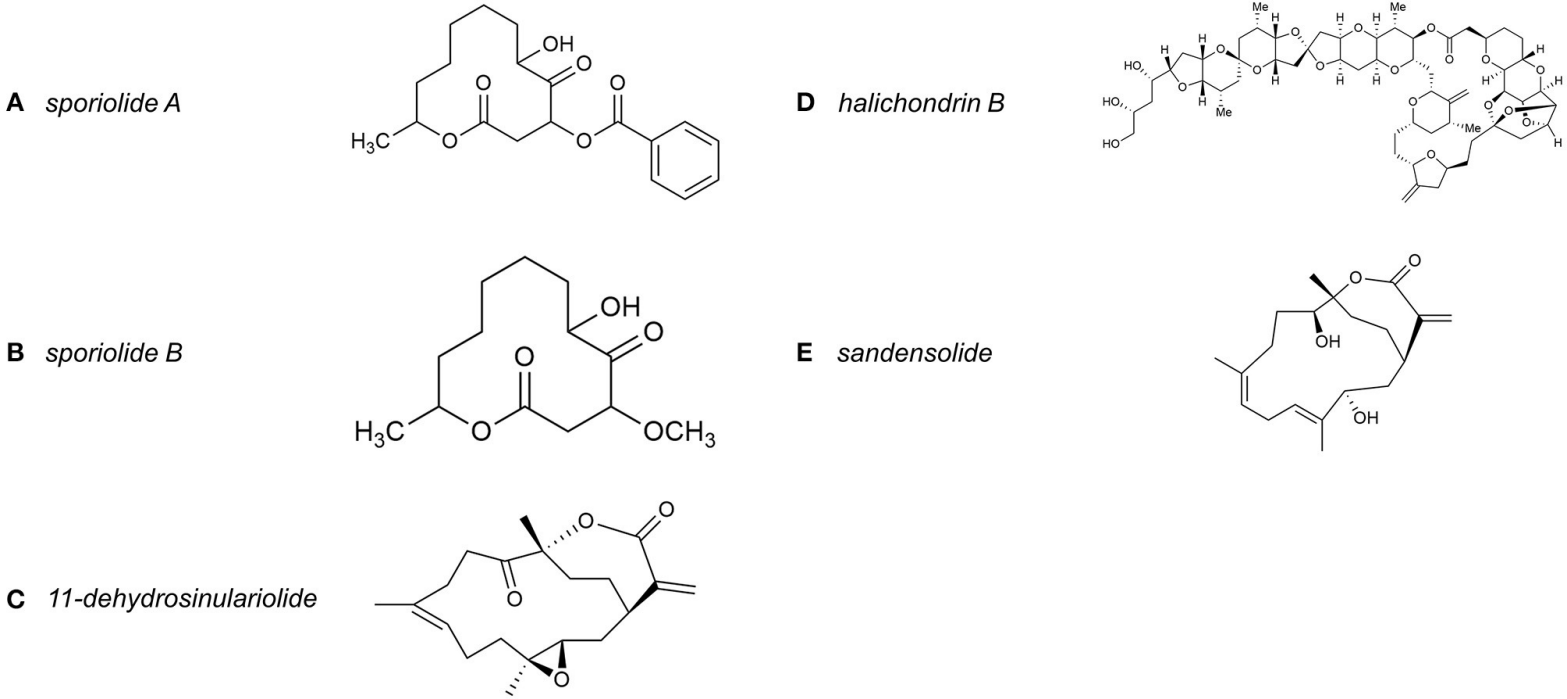
**(A–E)** The chemical structure of *halichondrin B, sporiolide A, sporiolide B, 11-dehydrosinulariolide*, and *sandensolide*.

Some marine algae contain bioactive compounds that are potential agents for OSCC treatment due to their ability to modulate the antioxidant defense system, apoptosis and autophagy in oral cancer (94). For instance, blue-green microalga *Spirulina* has been reported to reduce the risk of OSCC by reversing oral leukoplakias (97). In studies of anti-cancer effects, at least in part, by generating high levels of intracellular reactive oxygen species (ROS) (98). In the example of red algae, a methanolic extract of the red algae *Luminacin* (a marine microbial extract) induced autophagy and cell death in head and neck cancer cells (99, 100). Furthermore, a study by Yeh et al. (98) indicated that methanolic extract of *Gracilaria tenuistipitata* (MEGT) from seaweed extracts had apoptotic to oral cancer cells through DNA damage, ROS induction and mitochondrial depolarization. However, the above studies did not further address the anti-cancer mechanism of the specific bioactive components in the methanolic extract of the seaweed. Sporiolides A and B (Figures 7A,B) from the brown algae *Actinotrichia fragilis*, two new dodecameric macrolides, showed cytotoxicity against L1210 cells (101, 102). In addition, Zhang et al. (103) showed that fucoidan inhibited OSCC development by targeting filamin A (FLNA)-derived circular RNA (circFLNA) to mediate the expression of key proteins associated with cell growth, apoptosis, migration and invasion.

Bioactive components extracted from sea hare, sponges, corals, and fish also have the potential to modulate OSCC (104). A family of cyclic and linear peptides known as dolastatins as well as depsipeptides have been isolated from sea hare (*Dolabella auricularia*) and have been reported to be active anticancer components. Dolastatin-H (*Dolabella auricularia*) and Isodolastatin-H (*Dolabella auricularia*) have been shown to be highly cytotoxic agents (105, 106). Other cytotoxic agents such as polypropionate, auripyrone-A and B, were also isolated from *Dolabella auricularia* (105). Discodermolide, isolated from sponges (*Discodermia dissolute*), is the most promising natural product discovered to date. Discodermolide has been shown to be more effective than taxol and is being tested for the treatment of solid tumors (107, 108).

Another antitumor marine bioactive compound is 11-dehydrosinulariolide (an active ingredient isolated from the soft coral *Sinularia leptoclados*), which is also a potential antitumor bioactive ingredient (109). Liu et al. (109) noted that treatment for 6 h significantly induced early and late apoptosis in CAL-27 cells, providing a clue to the biochemical mechanism of the antitumor effect of 11-dehydrosinulariolide (Figure 7C) on CAL-27 cells. Bioactive components extracted from fish, such as Lejimalides (A-D), are unique 24-membered polyene macrolides isolated from a marine tunicate *Eudistoma cf. rigida*, exhibit potent *in vitro* cytotoxic activity (110). Pardaxin (H- GFFALIPKIISSPLFKTLLSAVGSALSSSGGQE-OH) is a 33 amino acid peptide, an antimicrobial peptide (AMP) isolated from marine fish species *Pardachirus marmoratus* (111–113). It is also a possible marine bioactive component for adjuvant chemotherapy in human OSCC care. Han et al. (114) pointed out that Pardaxin treatment caused cell cycle arrest in SCC-4 cells in the G2/M phase, thereby limiting cell proliferation.

Moreover, some cellular derivatives can be used to synthesize components with anticancer properties, such as E7389, a synthetic compound based on *halichondrin B* (Figure 7D), which was isolated from the Japanese sponge *Halicondria okadai* (115–117). Cheng et al. (118) investigated the effect of prodigiosin (PG), an alkaloid and natural red pigment as a secondary metabolite of *Serratia marcescens* on the growth inhibition of human oral squamous carcinoma cells. The results showed that PG induced autophagic cell death of OECM1 and SAS cells *in vitro* via the LC3-mediated P62/LC3-I/LC3-II pathway. This implied that PG might target the autophagic cell death pathway as a potential agent for cancer therapy. Furthermore, *sandensolide* (Figure 7E) isolated from *Sinularia flexibilis* also has an inhibitory effect on the growth of human OSCC cells, and its possible mechanism of action is the induction of oxidative stress-mediated cell death pathway (119).

## Challenges of Marine Bioactive Compounds in Oral Health

The ocean is naturally one of the richest sources of bioactive compounds that can be used as functional ingredients in the development of products for oral health benefits. These bioactive compounds can also be incorporated into dietary supplements, nutritional products and pharmaceuticals to exploit their potential health benefits. Currently, marine based nutraceuticals mainly include fish oil (mainly n-3 PUFAs), algal oil, shark liver oil and squalene, seaweed (macroalgae) and its components, chitin, chitosan (monomers), chitosan (oligomers), enzymes, peptides, protein hydrolysates and other products (120). Indeed, dietary factors, such as nutrients with antioxidant effects, immunomodulatory effects and those related to bone metabolism, have been shown to affect periodontitis directly or interactively (121–123). The highly diverse and dynamic nature of marine ecosystems has led to the identification of marine resource compounds as an important resource for potential functional foods (8, 9). However, despite the health potential of these compounds, their bioavailability affects their effectiveness in the human organism (124). Indeed, bioactive compounds whose complex properties and absorption mechanisms are such as metabolism, digestibility, and activity after absorption, are a scientific challenge to be further investigated and clarified.

For the development of oral health products, the maturity of the preparation process, clinical studies, safety doses and risks of marine bioactive ingredients should be considered for the industrialization of marine bioactive ingredients. For example, marine derivatives such as secondary metabolites are trace amounts and natural reserves are too small to sustain widespread use and development (101). The current marine ingredients with more mature preparation processes and production procedures are n-3 PUFAs, which are effective in alleviating a wide range of health conditions (125). Therefore, n-3 PUFAs related functional foods and special diets have been at the forefront of research and development. Meanwhile, the preparation process of sea cucumber polysaccharides has been also relatively mature, the separation and purification technology has been developed, and the biological activity has been studied (42, 126). However, research on the relationship between biological activity and conformation of sea cucumber polysaccharides is still lacking, and the theoretical support as a functional food ingredient needs further improvement. Although there are more and more supporters of fucoidan as functional food ingredients so far, the structure-function relationship of rockweed polysaccharides is still controversial (127–130). The structural backbone of most fucoidan is unknown, and the location and branching sites of their specific sulfate groups have not been described, leading to structure-activity relationships for fucoidan that have yet to be elucidated. Even though the broad biological activity of fucoidan has been demonstrated, there is a lack of pharmacokinetic data and their clinical application is still limited and needs to be further promoted (131, 132).

The stability of the bioactive substance is another important consideration for its potential application. This is because various functional components in food products play an important role in improving food properties and efficacy. Bioactive substances with health benefits such as probiotics, vitamins, minerals, polyphenols, n-3 polyunsaturated fatty acids are sensitive to oxygen, light, heat, water, pH, etc., which affects the shelf life of the food and the effective release during application. Alginates is an important microcapsule for loading bioactive ingredients in the preparation of functional foods, acting as a unique emulsification, thickening, gelation, film formation and other properties have important applications in encapsulating functional food factors (127). Xiao et al. (133) investigated the encapsulation rate and slow release effect of three ratios of calcium alginate, chitosan-encapsulated calcium alginate, and chitosan-calcium alginate direct mixture on sweet orange oil. It was found that the polymerization of calcium alginate and chitosan encapsulated sweet orange oil could control the prolongation of sweet orange oil during chewing of chewing gum to the maximum extent and prolong the flavor action time of chewing gum. Overall, marine compounds have outstanding health potential can be used as a health strategy to prevent or treat diseases and benefit the maintenance of oral health in humans. Marine biological ingredients have been used in food products, but further *in vivo* studies and human clinical trials as well as industrial promotion are needed for more marine biological active ingredients.

## Conclusion and Perspective

Oral diseases can cause pain, impaired function and reduced quality of life, resulting in considerable loss of productivity and financial burden to the patient. Dental health therefore has a direct impact on the normal life of people. Recent *in vitro* and *in vivo* studies have revealed the important role of marine bioactive components in the prevention of various oral health problems ranging from dental caries to halitosis and periodontal disease.

The studies mentioned in this review have shown that marine bioactive ingredients seaweed extracts, n-3 PUFAs, sea cucumber extracts, and marine bacterial metabolites have the ability to inhibit oral pathogens, repress their biofilms, and regulate the cancer cell cycle. Therefore, the marine bioactive ingredients mentioned in this paper such as n-3 PUFAs, sea cucumber extracts, and seaweed extracts can play a good role in inhibiting oral pathogenic bacteria, eliminating inflammation, and anti-tumor, which are good choices for developing oral functional foods, such as functional chewing gum or sugar-free tablets. However, before incorporating marine bioactive substances into functional food development, their industrial feasibility has to be evaluated and relevant studies have to be conducted to reveal the possible mechanisms of action of marine bioactive substances and long-term clinical trials.

As the global nutraceutical market grows, consumer interest in marine sourced ingredients is gradually increasing (10, 104). The potential applications of marine bioactive compounds in food, functional food and supplement development are receiving more attention. Compared to systemic diseases, oral diseases have a strong specificity and the vast majority of treatments require surgical operations for treatment, routinely contacting the patient's saliva, blood and gingival sulcus fluid. When patients carry respiratory-transmitted pathogens such as pneumonia virus, influenza virus, *mycobacterium tuberculosis* and other microorganisms in the oral cavity and nasopharynx, the aerosols and droplets produced can pose a serious hazard. Especially during this COVID-19 pandemic situation, many people undergoing treatment for dental diseases can be affected. Therefore, adequate oral hygiene is very important. Furthermore, with the rising awareness of oral disease prevention and dental health maintenance, functional foods and dietary supplements for oral health are favored by consumers.

In fact, a number of marine bioactive compounds have been used in the food industry as functional food ingredients to enhance the functional properties of foods or as additives to improve certain properties of foods (stability, emulsification, texture improvement). In the industrialization of marine bioactive ingredients, their bioavailability, purity, environmental friendliness, cost effectiveness, etc. are aspects that need attention. The studies on marine bioactive ingredients with mature preparation processes have been mostly focused on bioactivity exploration. Future research on two aspects of the marine bioactive ingredients is therefore recommended. On the one hand, the stability and adaptability of the activity need to be further investigated, that is, the chemical modification or organic synthesis of the structure, while preserving its physiological activity, in order to expand its application in the food field. On the other hand, the comprehensive utilization of low-value products/byproducts should be further undertaken. For example, the comprehensive utilization of polysaccharide-rich processing waste to cut down the cost of industrial applications. For the newly discovered marine biological active ingredients, further research on their functional structure, active mechanism of action, especially clinical application and even pharmacokinetic data support is needed. Furthermore, studies on health promotion, safe dosage and side effects of newly discovered compounds will also provide favorable theoretical support for their industrialization.

## Author Contributions

Y-ZH: writing the article. X-PD, L-BQ, and SS: critical revision of the article. Y-ZH, X-PD, B-WZ, ZJ, and Z-MW: final approval of the article. All authors contributed to the article and approved the submitted version.

## Funding

This work was supported by the National Key Research and Development Program of China (2019YFD0902000).

## Conflict of Interest

The authors declare that the research was conducted in the absence of any commercial or financial relationships that could be construed as a potential conflict of interest.

## Publisher's Note

All claims expressed in this article are solely those of the authors and do not necessarily represent those of their affiliated organizations, or those of the publisher, the editors and the reviewers. Any product that may be evaluated in this article, or claim that may be made by its manufacturer, is not guaranteed or endorsed by the publisher.
